# Effects of extreme mountain ultramarathon on stress‐related biomarkers from multiple biological matrices in adult male runners

**DOI:** 10.14814/phy2.70966

**Published:** 2026-06-04

**Authors:** Cristina Purcaro, Ester Sara Di Filippo, Alessandra Vezzoli, Danilo Bondi, Guido Giardini, Stefania Fulle, Vittore Verratti, Simona Mrakic‐Sposta

**Affiliations:** ^1^ Department of Neuroscience, Imaging and Clinical Sciences University “G. d'Annunzio” Chieti – Pescara Chieti Italy; ^2^ Institute of Clinical Physiology – National Research Council (CNR‐IFC) Milan Italy; ^3^ Mountain Medicine and Neurology Centre Valle D'Aosta Regional Hospital Aosta Italy; ^4^ Department of Science University “G. d'Annunzio” Chieti – Pescara Chieti Italy

**Keywords:** hypoxia, miRNA, noninvasive sampling, oxi‐inflammation, saliva, ultra‐endurance

## Abstract

Prior investigations concerning finishers of the Tor des Géants (TDG) have demonstrated oxi‐inflammatory response. MicroRNAs (miRNAs) have been identified as prospective biomarkers for oxi‐inflammatory and stress responses. This study addressed the acute responses concerning salivary miRNAs, circulating redox markers and urinary exercise‐induced markers during the 2019 TDG. It included seven healthy male participants who successfully completed the race. Biological specimens (blood, saliva, and urine) were collected 1–2 days prior to the race and immediately post‐completion, to assess redox system and glycemia from capillary blood, creatinine and neopterin concentrations from urine, and salivary hsa‐miR‐210 and hsa‐miR‐21. Circulating reactive oxygen species production increased, whereas total antioxidant capacity remained stable. Urinary creatinine and neopterin increased subsequent to the race. Nevertheless, salivary expression levels for both miR‐21 and miR‐210 displayed heterogeneity among participants. Variations in miR‐210 were significantly correlated with changes in heart rate. The extreme mountain ultramarathon incited cumulative stress reflective of muscle damage and immune response activation. Salivary miR‐21 and miR‐210 did not demonstrate acute alterations, indicating that they may not serve as highly responsive markers for the combined hypoxia‐strenuous exercise stressor; this finding may suggest the existence of adaptive mechanisms in finishers that facilitate their capacity to manage extreme challenges.

## 1 INTRODUCTION

When the human body is pushed towards its limits, biological processes manifest peculiarities that can be translated for medical and biological purposes. To capture the essence of nature–nurture conundrum, the field of exposomics has emerged as it comprehensively refers to chemical, biological, physical, lifestyle and social factors, as toxicants, pollutants, social threats, microbial invasions, viruses, temperature, oxygen, nutrients, physical activity, and psychological loads all contribute to the biological footprint and responses (Purcaro et al., 2024). Within this field, ultra‐sports can provide useful avenues for medical and biological investigations as they represent an extreme condition due to environmental stress, physical strenuous effort, and high psychological load. During an ultramarathon, runners typically experience dehydration with the decrease in body mass, occurrence of muscle pain, and acute‐phase pathological process in liver and kidney, accompanied by systemic and local inflammation (Knechtle & Nikolaidis, 2018).

Among the mountain ultramarathon circuit, the extreme “Tor des Géants” (TDG) consisted of 330 km with an altitude difference of 24,000 m, 25 mountain passes above 2000 m s.l., running in just one single stage across the four Giants of the Alps. The whole distance is divided into seven stages, with 35 aid‐relax stations providing opportunities for runners to rest and/or sleep. There are no established rules on rest and stops, the decision regarding when and how long to stop for rest and nourishment is made by the runner themselves. Previous results on TDG finishers revealed a marked oxi‐inflammatory response to the ultramarathon (Mrakic‐Sposta et al., 2015). Moreover, the analysis of urinary extracellular vesicles from experienced ultramarathon runners participating in the TDG revealed an increase in 5‐F2t‐IsoP after the race which increased after the race, thereby indicating the occurrence of a cellular oxidant injury (Biagini et al., 2024).

MicroRNAs (miRNAs) play a significant role in regulating oxi‐inflammatory and stress responses across various biological fluids, serving as potential biomarkers, raising the opportunity to identify more effective biomarkers of reversible and chronical disruption of homeostasis (Banerjee et al., 2017; Olejniczak et al., 2018). MicroRNAs (miRNAs) are endogenous short non‐coding RNA molecules that regulate gene expression at the post‐transcriptional level. Their expression changes in response to various physiological or pathological factors and are released into blood and other bodily fluids in a highly stable cell‐free form, making them useful biomarkers of several stress‐related responses and adaptations. Circulating miRNAs have been largely studied as acute and chronic biomarkers of endurance exercise, including ultramarathon (Eyileten et al., 2021); however, other biological fluids that are more readily sampled in field settings, such as saliva, may serve as a valid alternative for studying the acute response to ultramarathon stress at the level of miRNAs. More broadly, salivary biomarkers discovery is ever‐growing, since saliva represents an excellent source for obtaining biomarkers with simplicity and noninvasive sampling merits in comparison with other sources such as blood (Adeoye & Su, 2024). Indeed, the effects of high altitude exposure through salivary analyses have been previously carried out, for example, for microbiota, oxy‐inflammation, and several hormones (Jain et al., 2020; McLean et al., 1989; Pignatelli et al., 2024; Zhou et al., 2023). Among the stress‐related miRNAs, blood levels of miR‐21 has been linked to several stressors as physical exercise, hypoxia, inflammation, and aging (Horak et al., 2018); in particular, salivary miR‐21 can chronically increase due to inflammatory status, as oral lichen planus (Di Stasio et al., 2025), and acutely increase due to psychological stress reactions (Wiegand et al., 2018). MiRNAs also act as mediators of the hypoxic response, and miR‐210, ubiquitously expressed in broad range of cells, has emerged as the master hypoxamir (hypoxia‐inducible miRNA). In particular, high‐altitude environments remarkably influence human circulating miR‐210, which is associated to erythropoiesis (Yan et al., 2016); targets of miR‐210 involve plasticity and/or synaptic function as miR‐210 modulates the hypoxia‐induced neuroinflammation (Watts et al., 2018). It has been highlighted the role of miR‐210 as a hypoxia‐induced molecule: in particular, levels of miR‐210 in human saliva were similar to those in plasma and increased in response to hypobaric hypoxia (Yang et al., 2021). SalivaDB shows that miR‐21 has been largely used in diagnostic studies, particularly for oral disease, while studies on salivary miR‐210 are scarce (Arora et al., 2023).

### 1.1 Aims of the study

To provide novel insight into the physiological mechanisms involved under the extreme conditions of a mountain ultramarathon, this study focused on different biological fluids and biological systems. Possible associations between changes of salivary miRNAs, circulating redox markers, and urinary exercise‐induced markers were investigated. The hypothesis was that specific miRNAs linked to stress and hypoxia may prove to be salivary markers of the acute response to mountain ultramarathons, associated with typical responses of redox disorders and urinary markers linked to exercise‐related stress.

## 2 MATERIALS AND METHODS

### 2.1 Design and procedures

The study was performed during the 2019 edition of the TDG. During the pre‐race phase, a questionnaire was administered to collect personal data, and an assessment of healthy status of each participant was performed. Total body water (TBW) and fat mass (FM) were estimated by bioimpendentiometry (TBF‐300A Body Composition Analyzer; Tanita Corporation, Arlington Heights, IL, USA); blood pressure (BP) were acquired through a standard cuff sphygmomanometer (OMRON M7 Intelli IT, Omron, Japan); finger O_2_ saturation (SaO_2_) and Heart Rate (HR) were acquired through a pulse oximeter (Oximetry—Ohmeda TuffSat—GE Healthcare, Helsinki, Finland). These parameters were also attempted to be acquired immediately after the race, but the logistics strongly limited the possibility to register bioimpedance data in four participants, while only one missing value referred to post‐race weight.

These data were collected at rest. All participants were fully informed of the procedure before data collection, signed a written informed consent outlining study requirements. The procedure was conducted according to the Declaration of Helsinki. Ethical approval was obtained from the institutional Ethics Committee of the Aosta Hospital: Ospedale regionale “Umberto Parini”, Italy (Protocol n.2019.0088039I).

Participants were recruited on a voluntary basis, with enrollment supported by the Tor des Géants (TDG) organization prior to the event. The “Baseline” session (T0) was performed in Courmayeur 1 or 2 days before the race, depending on the availability of the athletes; participants followed at least 24 h of reduced activity/rest (Gussoni et al., 2023; Mrakic‐Sposta et al., 2015). The measures post‐race were immediately followed the end of the race in always in Courmayeur.

Capillary blood (100 μL) was taken from the fingertip: ROS production rate, antioxidant capacity, and glycemia were determined (Gussoni et al., 2023; Mrakic‐Sposta et al., 2021). Urine samples were collected by voluntary voiding in a sterile container before and after the race; samples were initially stored at 4°C in a portable cooler during the transport back to the laboratory, aliquoted, then stored at −80°C until assayed, and thawed only once before analysis (Giacon et al., 2022; Gussoni et al., 2023). Saliva samples were collected by asking participants to allow saliva to accumulate naturally in the mouth and directly into a sterile collection tube until the required volume of ~2 mL was reached; samples were initially stored at dry ice and transported back to the laboratory, then stored at −80°C until assayed, and thawed only once before analysis.

### 2.2 Participants

**FIGURE 1:**
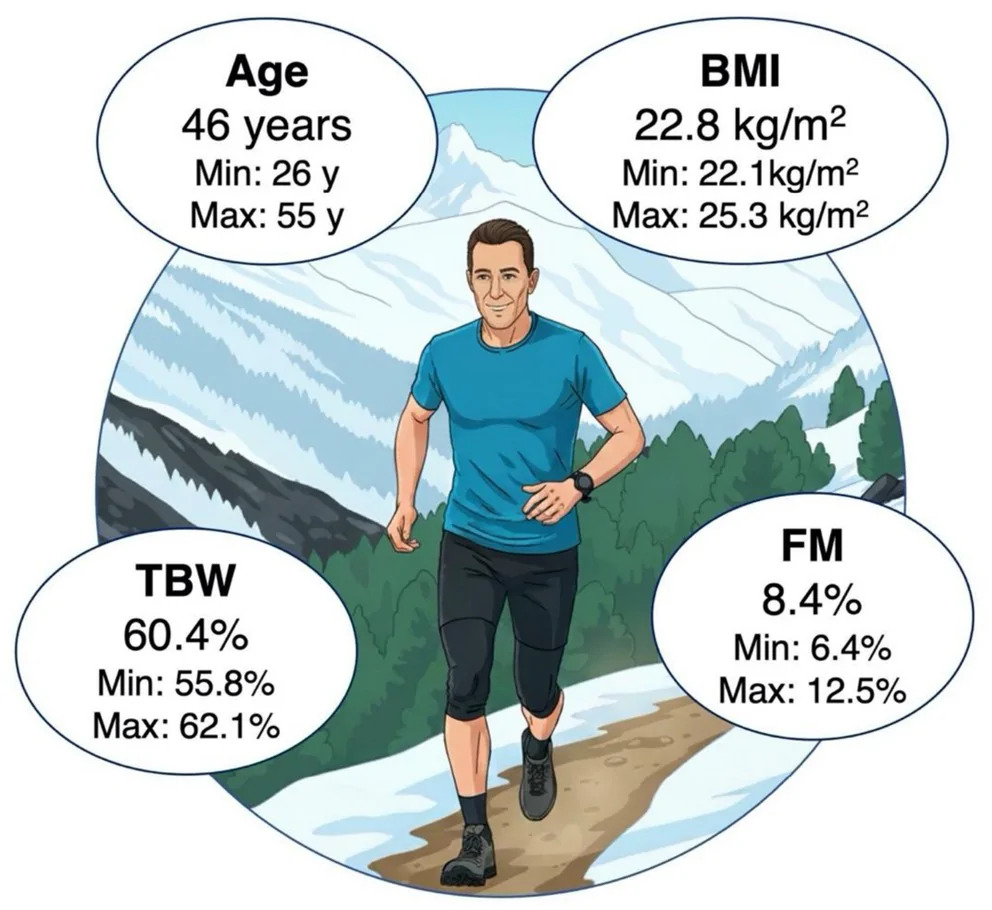
Characteristics of the seven ultra‐trail runners.

The final sample, the “Finisher of Tor des Geants” consisted of seven healthy males who had all successfully completed the TDG race in 2019. Their mean age was 43.57 ± 9.29 years, height was 177.00 ± 6.53 cm, weight was 73.03 ± 4.81 kg, and BMI was 23.27 ± 1.10 kg/m^2^. All of them previously participated in mountain races, and 6 out of 7 previously participated in ultra‐trail mountain races; their adherence to specific training ranged from half a year to 30 years. None of them reported cardiovascular or respiratory diseases, and only 1 was a smoker. They did not follow a specific dietary regimen, and only 2 reported intake of dietary supplements. Their pre‐race TBW was 60.11 ± 2.12% and their FM was 9.07 ± 2.28%. This subgroup (Figure 1) finely tuned the typical ultramarathoner, who is male with low body fat and aged ~45 years old (Knechtle & Nikolaidis, 2018).

### 2.3 Biomarkers

#### 2.3.1 ROS production

Electron paramagnetic resonance spectroscopy (EPR, X‐band, 9.3 GHz) (E‐Scan, Bruker Co., Billerica, MA, USA) was used to detect ROS production in capillary blood.

Capillary blood was immediately drawn after the race into lithium‐heparin capillary tubes; samples were immediately incubated with the CMH spin probe (1‐hydroxy‐3‐methoxy‐carbonyl‐2,2,5,5‐tetramethylpyrrolidine; Noxygen, Science Transfer & Diagnostics GmbH, Elzach, Germany) that reflects a global ROS burden and subsequently analyzed by EPR in real time.

A stable radical CP (3‐carboxy2,2,5,5‐tetramethyl‐1‐pyrrolidi‐nyloxy; Noxygen, Science Transfer & Diagnostics GmbH, Elzach, Germany) was used as an external reference to convert ROS determinations into absolute quantitative values (μmol·min^−1^). Methods were previously described (Gussoni et al., 2023; Mrakic‐Sposta et al., 2021). All samples were stabilized at 37°C using a Temperature Controller unit (Noxigen Science Transfer & Diagnostics GmbH, Elzach, Germany), interfaced with the spectrometer.

#### 2.3.2 Antioxidant capacity

Blood reducing capacity was assessed in 10 μL of capillary blood using a commercial EDEL electrochemical analyzer equipped with a redox sensor. The method is based on electrochemical oxidation of water‐soluble compounds within a defined potential range. A screen‐printed three‐electrode system (carbon working, counter, and Ag/AgCl reference electrodes) was used. The generated current was pseudo‐titrated to estimate biologically relevant antioxidants, and results were expressed in nW (Gussoni et al., 2023).

#### 2.3.3 Glycaemic test

Blood glucose levels were tested by the electronic device Glucose Accu‐Chek® with “Active” test strip (Roche diagnostic) on a drop of blood (Mrakic‐Sposta et al., 2015).

#### 2.3.4 Urinary biomarkers

Creatinine and neopterin concentrations were measured by an isocratic high‐pressure liquid chromatography (HPLC) method, as previously described (Mrakic‐Sposta et al., 2021).

#### 2.3.5 Salivary MicroRNA isolation and expression

Total miRNAs were isolated from saliva samples collected from subjects before and after the expedition. First, saliva samples were centrifuged at 16,100*g* for 20 min, and the supernatant was discarded. Total RNA, including miRNAs, was then extracted from the resulting pellet with the miRNeasy Micro Kit (#217084, Qiagen, Netherlands), following the manufacturer's instructions, and RNA concentration was quantified using a NanoPhotometer® NP80 (Implen, Germany). Subsequently, the RNA was reverse‐transcribed into complementary DNA through the High‐Capacity cDNA Reverse Transcription Kit (#4368814, Applied Biosystems, USA), according to the protocol provided by the manufacturer. Quantitative real‐time PCR (qRT‐PCR) was then performed in 96‐well plates using TaqMan™ miRNA probes and TaqMan™ Universal Master Mix II, no UNG (#4440040, Applied Biosystems, USA) with the QuantStudio™ 7 Pro Real‐Time PCR System (Thermo Fisher Scientific, USA). The following miRNA sequence probes (Applied Biosystems, Life Technologies, Italy) were used to assess miRNAs expression: hsa‐miR‐210‐3p (#000512), and hsa‐miR‐21‐5p (#000397). The hsa‐miR‐16‐5p (#000391, Applied Biosystems, USA) was used as a reference (Sullivan et al., 2022) to calculate the relative miRNA expression using the ΔCt method (*ΔCt = Ct*_miRNA of interest_−*Ct*_miR‐16_).

### 2.4 Data analysis

To compare pre‐ versus post‐race data, a series of paired samples Student's *t*‐tests was conducted, after checking the assumption of normality of residuals. Then, percent differences were calculated and the results were used to create pairwise correlations with both Pearson's parametric and Spearman's nonparametric methods; indeed, while tests for parametric assumptions were conducted, given the small sample size, both Pearson and Spearman correlation coefficients were calculated to ensure the robustness of the associations and to account for potential nonlinear relationships. Statistical significance was set for any *p* ≤ 0.05. Correlation *p* values were further subjected to the Holm‐Bonferroni step‐down method to control the family‐wise error rate across the unique pairwise comparisons. All analyses and graphs were conducted with Jamovi v. 2.3.21.0. Individual radar graphs were created with the AI tool Julius (https://julius.ai); radar graphs' levels were set exponentially (−1000%, −100%, −10%, 0, +10%, +100%, and +1000%) for a clearer appearance.

## 3 RESULTS

**FIGURE 2:**
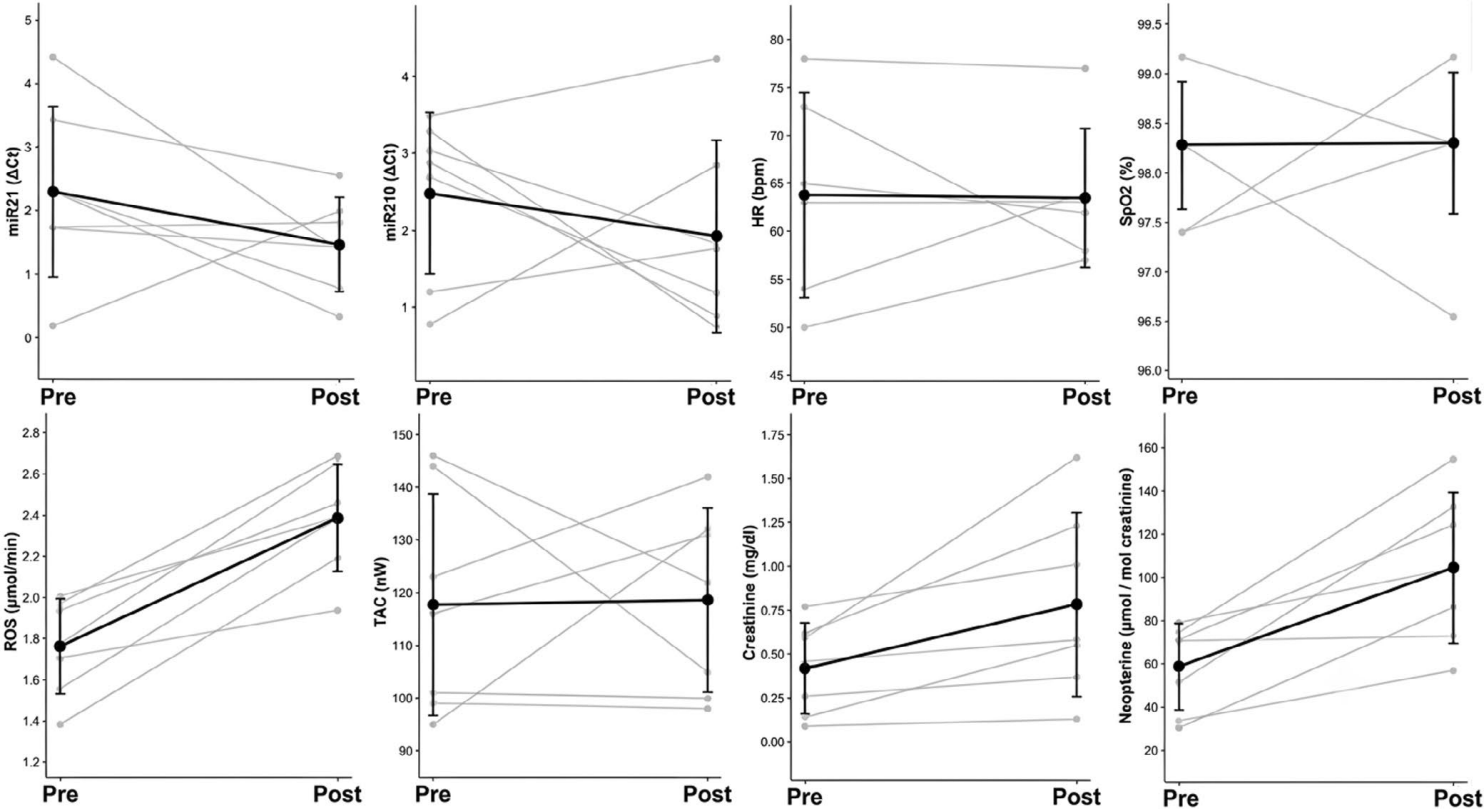
Pre‐post changes in variables from biological matrices; graphs were created with Google Colab and show all individual points along with mean and standard deviation.

Statistical pairwise comparisons (see Figure 2 and Table 1) revealed an almost significant reduction of weight after the race (from 73.90 ± 4.63 to 71.58 ± 4.71 kg); the changes of SBP and DBP (from 134.71 ± 17.43 to 138.17 ± 3.06 mmHg, and from 84.57 ± 7.30 to 84.50 ± 3.94 mmHg, respectively), as those of HR and SpO_2_ (from 69.86 ± 18.70 to 63.50 ± 7.18 bpm, and from 97.57 ± 0.98% to 97.83 ± 0.98%, respectively), were heterogeneous across participants, and no significant mean differences emerged (Table 1).

**TABLE 1:** Pairwise comparison of variables from pre to post‐TDG.

	Test	Statistic	df	*p*	Cohen's d	95% CI	
miR21	Student's *t*	1.412	6	0.208	0.534	−0.282	1.313
miR210	Student's *t*	0.866	6	0.420	0.327	−0.448	1.077
Weight	Student's *t*	2.481	5	0.056	1.013	−0.023	1.990
SBP	Student's *t*	−0.644	5	0.548	−0.263	−1.066	0.565
DBP	Student's *t*	0.302	5	0.775	0.123	−0.686	0.921
HR	Student's *t*	0.093	5	0.929	0.038	−0.764	0.837
SpO_2_	Student's *t*	0.000	5	1.000	0.000	−0.800	0.800
Glycemia	Student's *t*	−0.040	6	0.969	−0.015	−0.755	0.726
TAC	Student's *t*	−0.087	6	0.933	−0.033	−0.773	0.709
ROS	Student's *t*	−6.607	6	< 0.001	−2.497	−4.041	−0.918
Creatinine	Student's *t*	−2.736	6	0.034	−1.034	−1.944	−0.073
Neopterine	Student's *t*	−4.022	6	0.007	−1.520	−2.614	−0.376

Abbreviations: CI, confidence interval; DBP, diastolic blood pressure; HR, heart rate; SBP, systolic blood pressure; SpO_2_, peripheral oxygen saturation; ROS, reactive species of oxygen; TAC, total antioxidant capacity.

For what concerns circulating markers, ROS production rate increased after the race (from 1.82 ± 0.20 to 2.36 ± 0.23 μmol/min), while TAC did not (from 117.71 ± 21.09 to 118.57 ± 17.55 nW); glycemia remained very similar after the race (from 100.43 ± 14.89 to 100.71 ± 10.45 mg/dL). For what concerns urinary markers, creatinine increased after the race (from 0.42 ± 0.26 to 0.78 ± 0.52 mg/dL), as did neopterin (from 58.86 ± 20.24 to 104.61 ± 34.89 μmol/mol creatinine). For what concerns salivary markers, ΔCt of both miR‐21 and miR‐210 did not significantly change after the race (from 2.30 ± 1.34 to 1.46 ± 0.75, and from 2.48 ± 1.05 to 1.92 ± 1.24, respectively), as there was large heterogeneity across individual changes.

**FIGURE 3:**
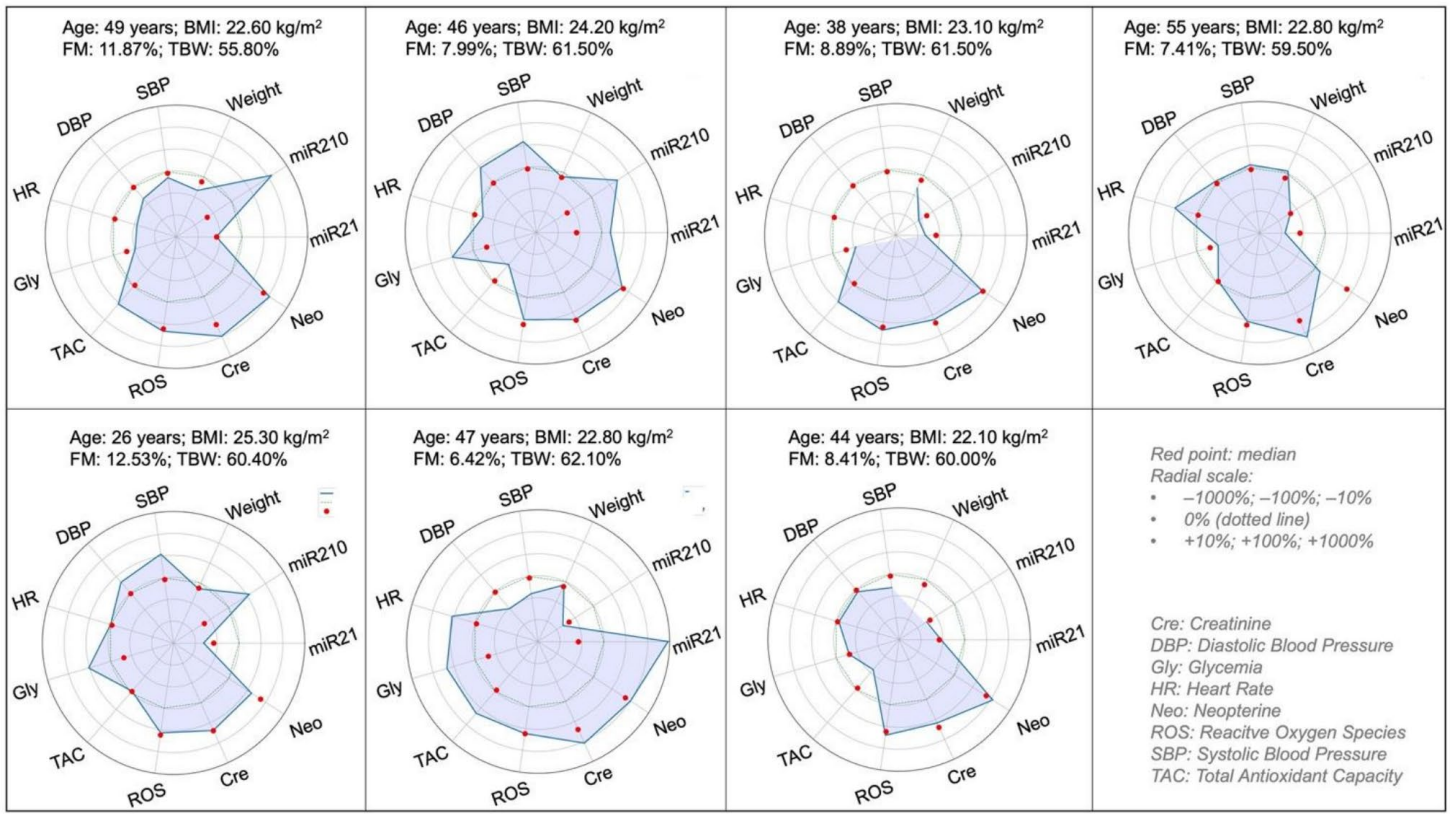
Radar graphs of individual changes.

A positive correlation was found between changes of miR‐21 with those of TAC, although non statistically significant (*p* = 0.086, *R* ^2^ = 0.478), and with those of glycemia (*p* = 0.022, *R* ^2^ = 0.684). Changes of miR‐210 were associated with those of HR (*p* = 0.023, *R* ^2^ = 0.761). Despite some coefficients were considered high, due to the conservative nature of the Holm‐Bonferroni correction and the small sample size, none of the correlation coefficients retained statistical significance after multiple testing adjustment. The other results of pairwise correlations are shown in Table 2, and individual changes are shown in Figure 3. Of note is the positive correlation between changes of ROS with those of neopterin.

**TABLE 2:** Pairwise correlations between percent differences from pre to post‐TDG.

		ΔmiR21	ΔmiR210	ΔWeight	ΔTAC	ΔROS	ΔCre
ΔmiR210	Pearson's *r*	−0.267	—				
	Spearman's rho	0.071	—				
ΔWeight	Pearson's *r*	0.213	−0.485	—			
	Spearman's rho	−0.029	−0.314	—			
ΔTAC	Pearson's *r*	0.691	0.055	−0.225	—		
	Spearman's rho	0.107	−0.143	−0.314	—		
ΔROS	Pearson's *r*	0.014	0.033	−0.825*	0.137	—	
	Spearman's rho	0.000	−0.464	−0.657	0.107	—	
ΔCre	Pearson's *r*	0.279	−0.109	0.693	0.366	−0.394	—
	Spearman's rho	−0.143	0.214	0.600	0.500	−0.536	—
ΔΝeο	Pearson's *r*	0.177	0.298	−0.705	−0.061	0.748	−0.440
	Spearman's rho	0.536	−0.107	−0.600	0.107	0.786*	−0.464

Abbreviations: Cre, creatinine; Neo, neopterin; ROS, reactive species of oxygen; TAC, total antioxidant capacity.

* None of the *p* values remained statistically significant after applying the Holm‐Bonferroni correction.

## 4 DISCUSSION

The extreme “Tor des Géants” exerted a cumulative stress on bodily systems, with the effects of this stress being evident in different body fluids and differentially interconnected. In comparison with the results obtained previously (Mrakic‐Sposta et al., 2015), it is evident that the extreme mountain ultramarathon resulted in a significant over‐production of ROS, accompanied by a substantially unchanged TAC. This was accompanied by an increased level of urinary creatinine and neopterin. In the aforementioned study, HR, SBP, and DBP increased at post‐race measurements; in contrast, in the present study, changes were more heterogeneous across participants. Glycemia remained on average unchanged in the two studies.

Creatinine and neopterin both increased in response to the TDG. Rather than being attributable to possible dehydration—as neopterin values were normalized to urinary creatinine—or renal dysfunction, the dual increase suggests cumulative muscle damage associated with immune response. This response is accompanied by a redox disturbance due to over‐production of reactive species in all TDG finishers, while the changes in systemic antioxidant capacity were heterogeneous across individuals. The higher the increase in systemic ROS, the higher the urinary level of neopterin; this suggests an integrated response of immuno‐oxi‐inflammation due to the extreme stress of the mountain ultramarathon. Monocytes and macrophages are the most relevant sources of neopterin, which is involved in the activation of the redox‐sensitive transcription factor NF‐κB, thus enhancing the production of ROS (Gostner et al., 2013).

We hypothesized a multifaceted stress response after TDG, which included putative salivary miRNAs along with altered redox networks and urinary exercise‐induced markers. Here, both salivary miRNAs' expressions remained similar between pre‐ and post‐race samples. However, reduction of miR‐21 expression was associated with increasing TAC and glycemia, while reduction of miR‐210 was associated with increasing HR, highlighting a possible systemic response that may emerge in spite of the absence of individual variables' mean changes. In this study, salivary miR‐21 changes did not mirror those expected in response to several stressors as physical exercise and hypoxia (Horak et al., 2018); nor did the master hypoxamir miR‐210, which instead was previously observed to change in human saliva similarly to plasma in response to hypobaric hypoxia (Yang et al., 2021). Consequently, the hypothesis that miR‐21 and miR‐210 in saliva are highly responsive markers of a combined hypoxia‐strenuous exercise stressor, such as that of TDG, cannot be concluded.

The potential influence of weight loss following the race on the results regarding analytes concentrations in biological fluids cannot be discounted; however, it is considered that the expression of miRNAs in saliva is not directly dependent on the decrease in body mass. It is also possible that variations in training levels may have had a role to play in accounting for individual variations; in any case, given the nature of the race, all participants were evidently fit enough to sustain an extreme aerobic performance such as the TDG.

It is conceivable that, in conjunction with the substantial physiological changes resulting from TDG, there exist adaptive mechanisms in TDG finishers that enable them to cope with extreme challenges, thereby serving as protective measures, as evidenced elsewhere by the anticipatory pacing strategy and mechanical adaptations (Degache et al., 2016; Saugy et al., 2013). Therefore, a comparatively elevated stress response and multifaceted interconnectedness may manifest in TDG non‐finishers; it is possible that a further step of analysis during the race could confirm the hypothesis that integrated stress is more pronounced in those who do not finish the race.

## AUTHOR CONTRIBUTIONS

**Cristina Purcaro:** Conceptualization; investigation; methodology. **Ester Sara Di Filippo:** Project administration; resources; supervision. **Alessandra Vezzoli:** Investigation. **Danilo Bondi:** Conceptualization; formal analysis; visualization. **Guido Giardini:** Project administration; resources; supervision. **Stefania Fulle:** Resources; supervision. **Vittore Verratti:** Resources; supervision. **Simona Mrakic‐Sposta:** Conceptualization; investigation; methodology; project administration; resources; supervision.

## FUNDING INFORMATION

This research received no specific grant from any funding agency in the public, commercial, or not‐for‐profit sectors.

## CONFLICT OF INTEREST STATEMENT

The authors declare that there is no conflict of interest.

## ETHICS STATEMENT

Ethical approval was obtained from the institutional Ethics Committee of the Aosta Hospital: Ospedale regionale “Umberto Parini”, Italy (Protocol n.2019.0088039I).

## CONSENT

All participants were fully informed of the procedure before data collection, signed a written informed consent outlining study requirements.

## Data Availability

The data that support the findings of this study are available from the corresponding author upon reasonable request.
